# In Situ Nanoscale Dynamics Imaging in a Proton‐Conducting Solid Oxide for Protonic Ceramic Fuel Cells

**DOI:** 10.1002/advs.202202096

**Published:** 2022-06-24

**Authors:** Oleg Gorobtsov, Yumeng Song, Kevin Fritz, Daniel Weinstock, Yifei Sun, Dina Sheyfer, Wonsuk Cha, Jin Suntivich, Andrej Singer

**Affiliations:** ^1^ Department of Materials Science and Engineering Cornell University Ithaca NY 14853 USA; ^2^ X‐ray Science Division Advanced Photon Source Argonne National Laboratory Lemont IL 60439 USA

**Keywords:** ceramics, energy materials, fuel cells, hydrogen, materials science

## Abstract

Hydrogen fuel cells and electrolyzers operating below 600 °C, ideally below 400 °C, are essential components in the clean energy transition. Yttrium‐doped barium zirconate BaZr_0.8_Y_0.2_O_3‐d_ (BZY) has attracted a lot of attention as a proton‐conducting solid oxide for electrochemical devices due to its high chemical stability and proton conductivity in the desired temperature range. Grain interfaces and topological defects modulate bulk proton conductivity and hydration, especially at low temperatures. Therefore, understanding the nanoscale crystal structure dynamics in situ is crucial to achieving high proton transport, material stability, and extending the operating range of proton‐conducting solid oxides. Here, Bragg coherent X‐ray diffractive imaging is applied to investigate in situ and in 3D nanoscale dynamics in BZY during hydration over 40 h at 200 °C, in the low‐temperature range. An unexpected activity of topological defects and subsequent cracking is found on a nanoscale covered by the macroscale stability. The rearrangements in structure correlate with emergent regions of different lattice constants, suggesting heterogeneous hydration. The results highlight the extent and impact of nanoscale processes in proton‐conducting solid oxides, informing future development of low‐temperature protonic ceramic electrochemical cells.

## Graphical abstract

Proton‐conducting ceramics open new horizons for hydrogen energy devices in the lower operating temperature ranges, promising higher energy efficiency. Nevertheless, nanostructure plays a more important role in lowering the operating temperature. Presented here are the results of in situ X‐ray imaging of nanostructure dynamics in BaZr_0.8_Y_0.2_O_3‐d_ during hydration, exposing a unique degree of freedom in proton‐conducting solid oxide materials.

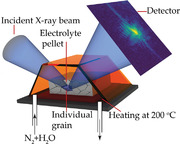

## 1 Introduction

The drive for clean and energy‐efficient electrochemical devices for hydrogen energy has brought much attention to high proton‐conducting solids, from low‐cost proton‐conducting polymers to more chemically and mechanically stable proton‐conducting ceramics (PCCs).^[^ ^1^, ^2^ ^]^ PCCs are unique solid electrolytes that acquire protons from ambient hydrogen and water vapor through equilibration with oxide lattice defects.^[^ ^2^, ^3^ ^]^ Efficient proton transport in PCCs has allowed solid‐state electrochemistry at temperatures below 600 °C or even 400 °C, making them attractive for electrochemical energy conversion and electrochemical manufacturing. To date, PCCs have shown promise for a wide range of technological applications, such as fuel cells for energy conversion^[^ ^4^, ^5^, ^6^ ^]^ and membrane reactors for hydrogen production.^[^ ^7^, ^8^ ^]^ Further advances in the performance and reliability of protonic ceramic electrochemical devices rely on improvements in the long‐term transport properties and structural stability of the proton‐conducting solid oxide electrolytes in the intermediate‐ and low‐temperature range.^[^ ^9^, ^10^ ^]^

**Figure 1:**
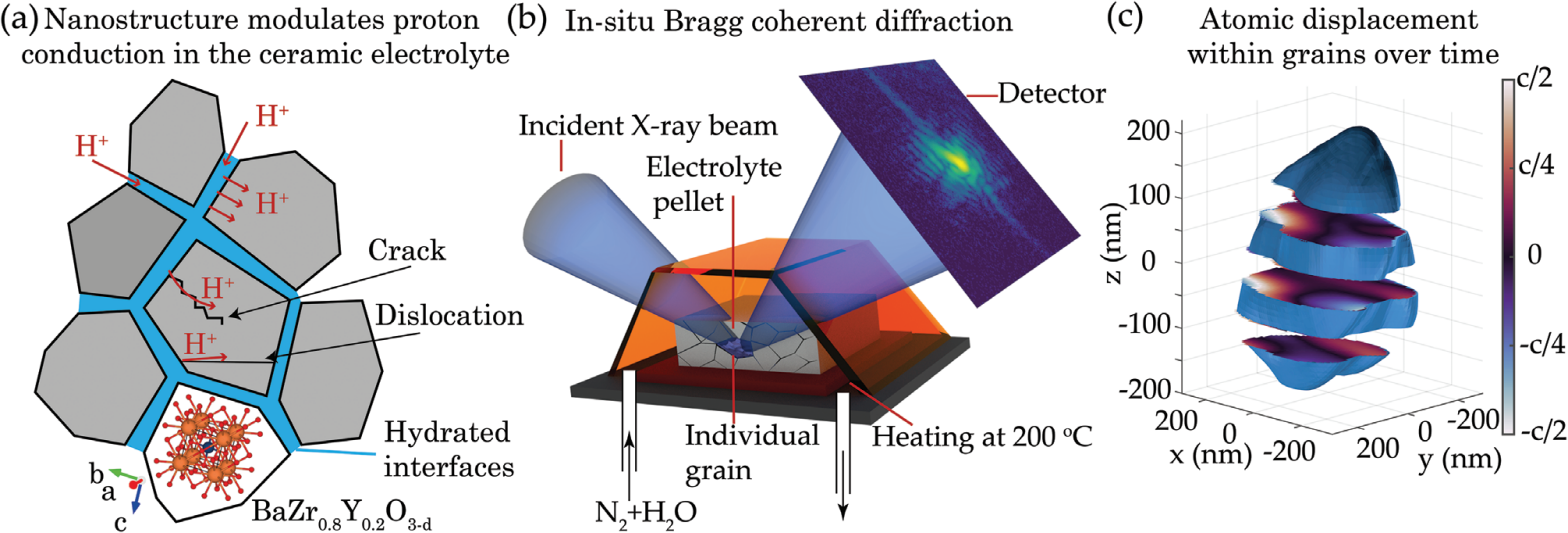
a) Defects in the crystal structure, grain boundaries, and cracks change the proton conduction of the bulk material. b) Incident coherent X‐ray beam produces speckled diffraction patterns from individual grains in the polycrystalline electrolyte pellet. While the X‐ray beam illuminates many grains, the angular sensitivity of Bragg scattering allows us to isolate signal from individual crystalline grains embedded in the pellet. The heating element maintains temperature while N_2_/H_2_O is pumped through the chamber. The pellet is rotated in the beam, producing a 3D diffraction peak, c). The diffraction peak can either be analyzed directly or used to retrieve the 3D particle shape and displacement field.

Acceptor‐doped barium zirconate, particularly the yttrium‐doped barium zirconate BaZr_0.8_Y_0.2_O_3‐d_ (BZY) is a highly chemically stable perovskite oxide with excellent proton conductivity below 600 °C, making it a popular candidate for hydrogen fuel cells, electrolyzers, and electrochemical synthesis.^[^ ^8^, ^11^, ^12^, ^13^, ^14^ ^]^ In polycrystalline PCCs and BZY in particular, nanostructure, including grain boundaries and defects, can increase the protonic resistance or adjust proton conductivity and lower the operating temperatures down to 400 °C or less (**Figure** **1**a).^[^ ^6^, ^15^, ^16^, ^17^, ^18^, ^19^ ^]^ The interfacial area between grains can stabilize the interfacial hydrated layer to provide a pathway for protonic conduction in the polycrystalline BZY.^[^ ^18^ ^]^ The mechanism is available at low temperatures typically not higher than 200 °C. Static strain and misfit dislocations also demonstrably affect electrochemical performance.^[^ ^20^, ^21^ ^]^ The structural instability during operation can affect the performance of the protonic ceramic electrochemical cells, as the crystal structure changes modulate proton conduction.^[^ ^9^ ^]^

Recent in situ studies have widely examined chemical processes in BZY^[^ ^12^, ^22^, ^23^, ^24^ ^]^ and macroscale or averaged structure, but the nanoscale dynamics in proton‐conducting solid oxide electrolytes and electrodes remain largely unstudied. Intra‐ and inter‐particle stresses developed from chemical processes and the proton transport mean that the material grains will eventually develop defects, strain gradients, and cracks, changing the macroscopic properties. Still, the lack of in situ characterization on the nanoscale has prevented definitive conclusions about these processes' timescale and extent. To understand the nanostructure dynamics, one must extract information about strain, defects, and crystal coherence within individual submicron‐sized grains embedded in a polycrystalline, 10–1000 micron thick material. The unique problem is to achieve a sub‐100 nm resolution while imaging in 3D defects and shape of a single grain surrounded by millions of similar grains, despite high absorption and hazardous operating conditions. For these reasons, imaging the evolving structure with sufficient resolution in situ with electron microscopy is challenging.^[^ ^2^ ^]^ X‐ray^[^ ^25^ ^]^ and neutron^[^ ^26^ ^]^ diffraction methods, in comparison, provide sufficient penetration depth for in situ structure investigations in ceramic materials but conventionally only provide averaged information on the structure over multiple crystal grains and inadequate spatial resolution.^[^ ^2^ ^]^

Recently, Bragg coherent X‐ray diffraction Imaging (BCDI)^[^ ^27^, ^28^, ^29^, ^30^ ^]^ has enabled operando imaging of the nanostructure dynamics in battery materials, where similar limitations exist. BCDI is performed at high intensity coherent sources of X‐ray radiation such as synchrotrons and free electron lasers. An X‐ray beam focused within a polycrystalline material produces an X‐ray scattering peak from a crystal grain in the beam only when the crystallographic orientation of the grain and the beam direction satisfy Bragg condition. Therefore, scattering from different grains can be separated and selected by moving and/or rotating the material. Coherent scattering from individual grains in polycrystalline electrolytes and electrodes produces speckle patterns uniquely dependent on the internal structure and shape of the grain (example in Figure 1a). BCDI requires no spatial scanning and uses beam size comparable to the grain size. The spatial resolution is defined by the diffraction pattern. Phase retrieval^[^ ^30^, ^31^ ^]^ on a 3D Bragg peak collected by rocking the sample provides the 3D structure of the grains and the atomic displacement within (Figure 1b), reaching sub‐100 nm resolution for strain and particle shape, and detecting non‐equilibrium defects such as dislocations and domain boundaries.

Here, we used the grain Bragg coherent X‐ray diffractive imaging (gBCDI)^[^ ^32^ ^]^ to track in situ the evolution of nanostructure and defects within the individual grains in the polycrystalline BZY. gBCDI enables nanoscale imaging of the changes in atomic displacement and defects dynamics in submicron‐sized grains within a polycrystalline material, going far beyond incoherent X‐ray diffraction capabilities. We combined direct analysis of coherent diffraction and gBCDI to track nanostructure evolution within BZY on an individual grain level at 200 °C during hydration. Despite macroscale stability, we find that multiple non‐equilibrium structural defects and new grain facets develop on a timescale of hours, despite 200 °C belonging in a low operating temperature range for BZY electrochemical devices. We find clear evidence of the grain separating into smaller coherent volumes directly visible in the reciprocal maps of multiple X‐ray Bragg peaks. We demonstrate that the dynamical evolution and nucleation of topological defects and subsequent grain cracking are present on the nanoscale in BZY during hydration even at 200 °C, and find that unique crystal facets appear in non‐equilibrium conditions. Clearly separable regions of different crystal lattice constants, suggesting inhomogeneous hydration, develop in unison with the appearance of defects and cracking. Nanostructure dynamics must be controlled to make protonic ceramic electrochemical devices viable and efficient.

## 2 Results and Discussion

### 2.1 Evidence of Nanostructure Dynamics in Coherent X‐Ray Scattering

**Figure 2:**
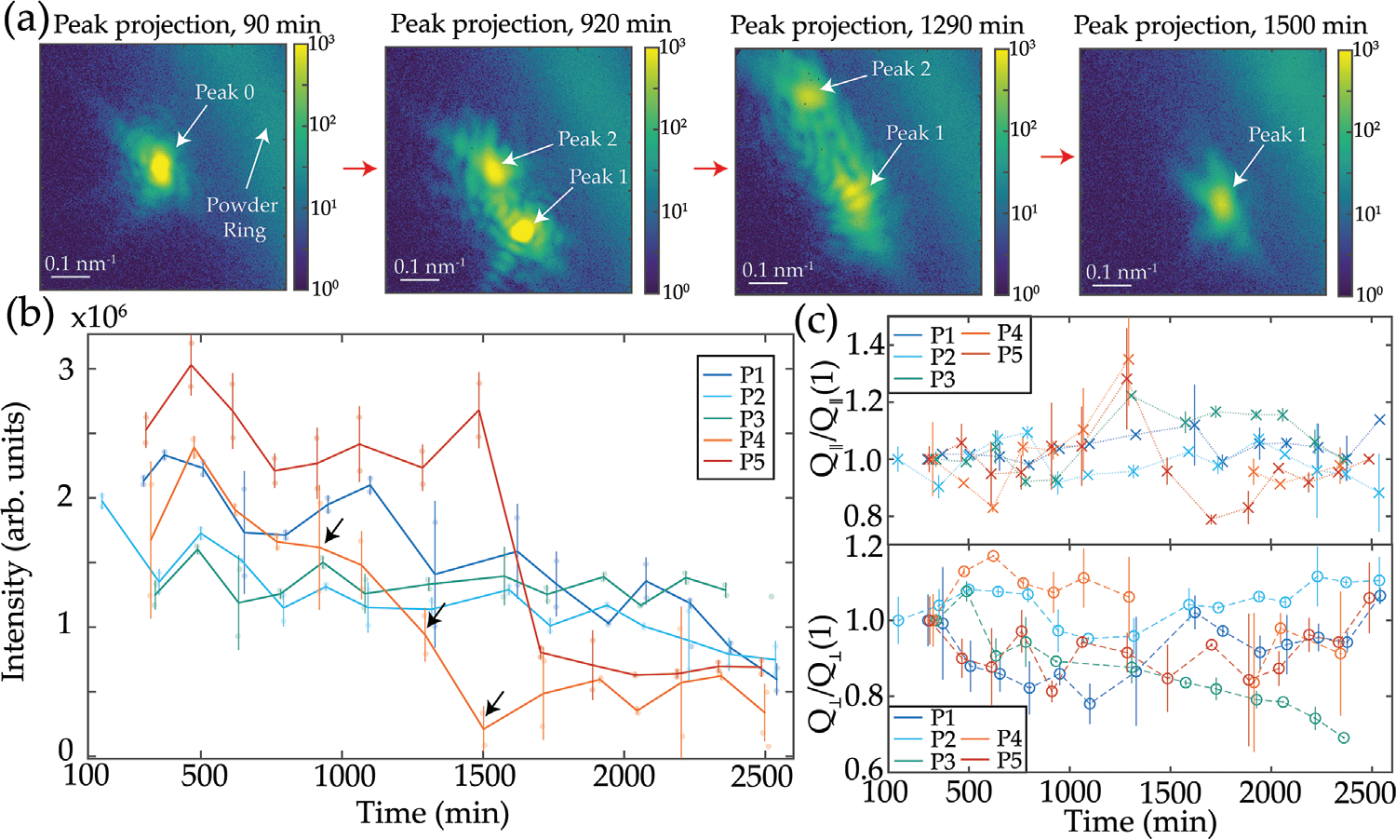
Direct Bragg coherent scattering analysis. a) Example of a Bragg peak splitting during the performed in‐situ measurement. Grain label P4. b) The total intensity of a Bragg peak for different grains as a function of time. Every data point represents a unique condition of an individual grain at a given time (*n* = 1). Error is estimated as the difference between two measurements with 2 min delay. c) Evolution of the relative peak width parallel (top) and perpendicular (bottom) to the scattering vector for different grains. Every data point represents a unique condition of an individual grain at a given time (*n* = 1). Peak width is estimated as standard deviation *σ* of the intensity distribution, error is estimated as an error on standard deviation $\sqrt[{4}]{\mu_{4}-\sigma^{4}}$.^[^ ^33^ ^]^

We performed a coherent X‐ray diffraction experiment on the polycrystalline BZY sintered at 1100 °C (details on synthesis in Experimental Section and pre‐characterization in Supporting Information). We collected (110) Bragg diffraction peaks from individual grains within the sintered pellet for over 30 h at 200 °C, while a humid nitrogen atmosphere was pumped through the chamber (setup scheme in Figure 1b). Analysis of the reciprocal space maps from the grains provides immediate information on the comparative structural evolution of multiple grains (**Figure** **2**). Before the real‐space imaging with phase retrieval, significant changes in the diffraction patterns are already noticeable. We see splitting and separation of a single Bragg diffraction peak into multiple peaks (Figure 2a) over several hours. The angular separation between the splitting peaks grows initially with a speed of ≈0.5 mrad h^−1^ before the separation rapidly increases. At this point, the changes in separated diffraction peaks can no longer be followed simultaneously. Splitting occurs mainly perpendicular to the scattering vector q. The remaining total scattered intensity in the brighter Bragg diffraction peak after complete separation is two to three times smaller than the intensity before the split, showing a steep decrease in the crystal coherent volume within the grain (Figure 2b, grains P4 and P5).

The peak splitting suggests that the crystal grain splits initially into slightly misaligned domains, producing separated by ≈1 mrad scale angle but still simultaneously visible peaks. Subsequently, growing misorientation suggests the fracture of a single grain into two different grains of a smaller individual volume. Figure 2b shows divergent behavior among measured grains, suggesting that local environment and grain form factor affects the degree and timing of coherent volume loss (responsible for intensity drop). Because diffraction of both domains in Figure 2a is visible while illuminated with an ≈1 µm X‐ray beam, the domains remain close. The angular splitting is due to relative angular misorientation between the domains. The full separation of the peaks after several hours supports the idea of fracture, and not domains with different hydration. The slow (over several hours) speed of the misalignment is explained by the restrictions imposed by the neighboring grains in the sintered pellet. To further investigate the structural deformation during fracture, we have investigated the evolution of the diffraction peak widths during and after crack propagation. The width of a diffraction peak can serve as a proxy for the coherence of the crystal structure. Degradation of the crystal structure commonly presents itself in the growing average strain and number of defects in the grains, increasing the Bragg peak width. Notably, the Bragg peak width both along and perpendicular to the scattering vector q (Figure 2c) does not demonstrate a preferred increase or decrease of the width over the different grains. However, while particles 4 and 5 in (Figure 2c) clearly tend to increasing peak width up to 40% parallel to q (thus not caused simply by peak splitting, which happens perpendicular to q), signifying an increase in strain and/or defects, the peak width decreases rapidly after the peaks entirely separate. The decrease in peak width after the cracking suggests that the grain cracking relieves stress and non‐equilibrium defects in the grain. The absence of lingering strain gradient suggests brittle fracture with no significant permanent structural rearrangements away from the crack surface. The rest of the particles present diverging behavior, with peak width variation within 10–20% higher or lower than the pristine state. It is important to note that peak width perpendicular to Q is inaccessible by conventional X‐ray Diffraction (XRD), in which diffraction structure over the direction perpendicular to q is averaged out and is thus insensitive to the peak splitting observed here.

### 2.2 3D Imaging of the Cracking Process

**Figure 3:**
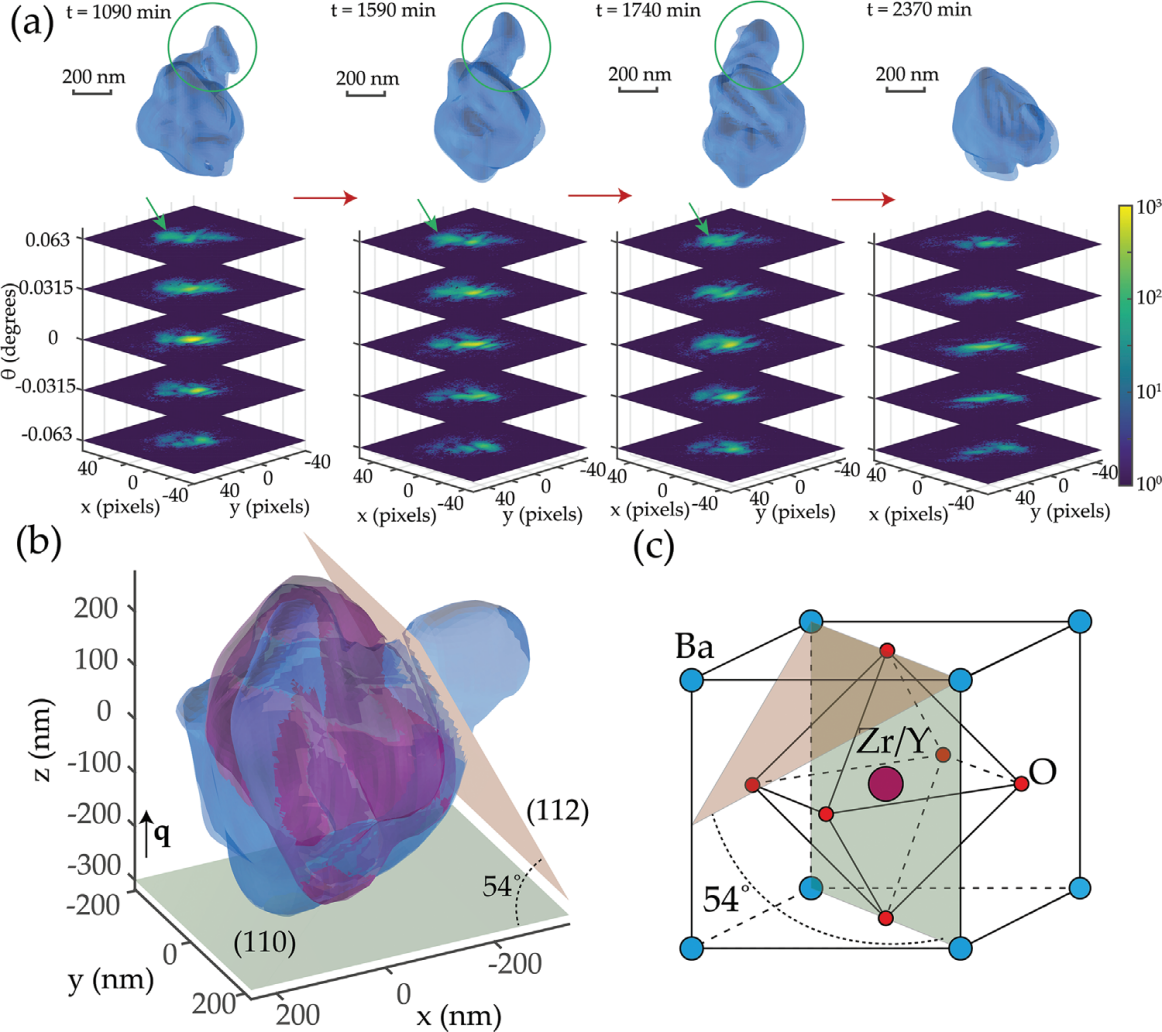
Grain shape evolution. a) Example of the changes in the Bragg peak and the changes in shape for a reconstructed grain (P2 in Figure 3). Isosurface at 15% maximum amplitude, slight additional variation in form is due to the uncertainty in the modulus of the retrieved complex amplitude. b) Example of the cracking. Blue surface—particle shape at *t* = 1590 min, magenta surface—particle shape at *t* = 2370 min, green horizontal plane—(110) crystallographic plane, brown plane—(112) crystallographic plane. c) BaZr_0.8_Y_0.2_O_3‐d_ unit cell schematic with (110) and (112) planes marked.

We further investigated the evolution of the shape and internal structure of the grains by performing phase retrieval^[^ ^30^ ^]^ on the collected Bragg diffraction peaks. We have successfully retrieved in three dimensions the shape of and the atomic displacement field within grains at specific times during the 30 h period. The grain size of all measured grains ranged from 0.5 to 2 µm. Interestingly, even when the total scattering intensity decreases only by 10–20% and without apparent peak separation, as in grain P2 in Figure 2b, a sharp change in the grain shape consistent with cracking is visible (example in **Figure** **3**a, top). The grain of ≈500 × 500 × 500 nm size changes shape at ≈2000 min into the in situ measurement. Part of the volume present at 1000–1800 min, marked by a green circle in Figure 3a, disappears at 2370 min and beyond, signifying the loss of crystal coherence with the rest of the grain. In the 3D coherent Bragg peak itself, the change is accompanied by a disappearance of a satellite maximum (marked by green arrows in Figure 3a, bottom). Overlapping reconstructed grain shapes at 1590 min and at 2370 min (Figure 3b) confirm the disappearance of a crystal volume. The comparison of the shape before and after fracture allows us to determine the orientation of the crack plane in comparison to the scattering vector q∥z, oriented normal to a crystallographic plane from {110} family (green). We find an angle of ≈50–60 degrees, most closely matching to a plane from {112} family (brown). (112) crystallographic plane is oriented at an angle 54 degrees to (110) plane in the BZY perovskite crystal structure (Figure 3c).

In equilibrium, dominant facets of BZY crystals are along {001} and {110} plane families.^[^ ^34^, ^35^, ^36^ ^]^ In BZY nanocrystals, {111} facets have been observed.^[^ ^37^ ^]^ Therefore, cracking along the {112} plane family, leading to {112} facets, is unexpected. Previously, similarities between BZY and CeO_2_ nanocrystals have been found,^[^ ^37^ ^]^ and in CeO_2_ {112} planes are possible termination planes,^[^ ^38^ ^]^ although they spontaneously turn into a stepped {111} surface. The resolution of our measurement is insufficient to observe a surface rearrangement to a stepped surface; however, to the authors' knowledge, no {112} termination planes have been previously reported in BZY.

### 2.3 Topological Defect Nucleation

**Figure 4:**
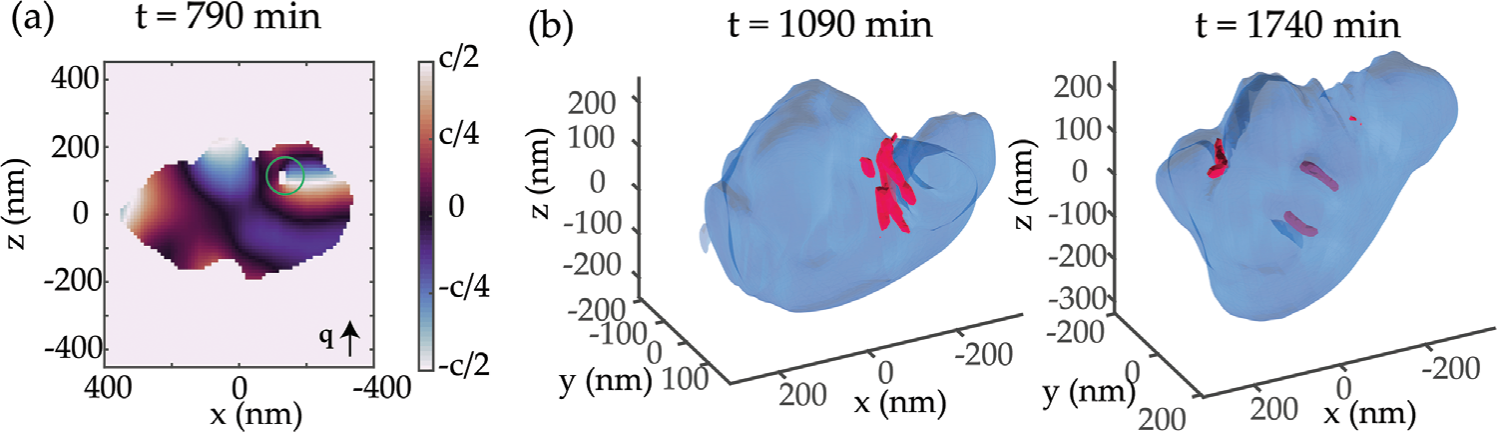
Typical dislocations nucleating in the grain. a) Sample cross‐section of a reconstructed displacement inside a grain, showing a vortex and a drop in amplitude (green circle) signifying a dislocation. In 3D, the dislocation line (red) goes through the grain (blue). b) Dislocation lines (red) in the grain P2 (blue) on the border of the cracking region.

Furthermore, the complex phase of the real‐space complex amplitude retrieved through gBCDI provides in situ information on the 3D distribution of atomic displacement within the grains in the [110] direction. Analysis of the atomic displacement within the P2 grain demonstrates the abundance of dislocations generated during the in situ process (**Figure** **4**). Dislocations with a component of the Burgers vector b along the scattering vector q produce a singularity in the atomic displacement. They can be pinpointed as vortices in the displacement field (Figure 4a, marked by a green circle), also producing zeroes (“holes”) in the retrieved shape (see the center of the vortex in Figure 4a) because of undefined displacement at the dislocation core. We pinpoint the dislocation lines in 3D (Figure 4b, red lines) by tracking the singularities in the retrieved displacement through the grain. Multiple dislocations with different orientations of the dislocation line are found in the grain P2, evolving over time. Interestingly, the grain volume that later detaches demonstrates a particular proclivity for dislocations (Figure 4b). Note the jagged appearance of the grain surface in the region due to the zeroes in amplitude produced by dislocations. While the orientation of the dislocation lines differs, all of them have a component in the (110) plane, perpendicular to the scattering vector q. Note that a screw dislocation with a dislocation line entirely in the (110) plane would not produce a vortex in the atomic displacement because the Burgers vector would be oriented perpendicular to the scattering vector q||[110], which suggests that the dislocations are preferentially of the edge or mixed type. Additionally, our experimental geometry is only sensitive to dislocations with the Burgers vector not perpendicular to the Q vector, suggesting there might be more dislocations we do not see in the displacement field.

While perovskites do not form an isomechanical group, in perovskites such as SrTiO_3_ and KNbO_3_, and theoretically generally in perovskite oxides, edge dislocations aligned along <110> at low temperatures (<1000 K) are mobile and dissociate producing stacking faults.^[^ ^39^, ^40^ ^]^ Our in situ imaging results show that the dislocation configuration changes at a sub‐hour timescale in BZY, showing experimentally similar <110> dislocation behavior to the one theoretically predicted for other oxide perovskites.

### 2.4 Strain Distribution: Evidence of Inhomogeneous Hydration

**Figure 5:**
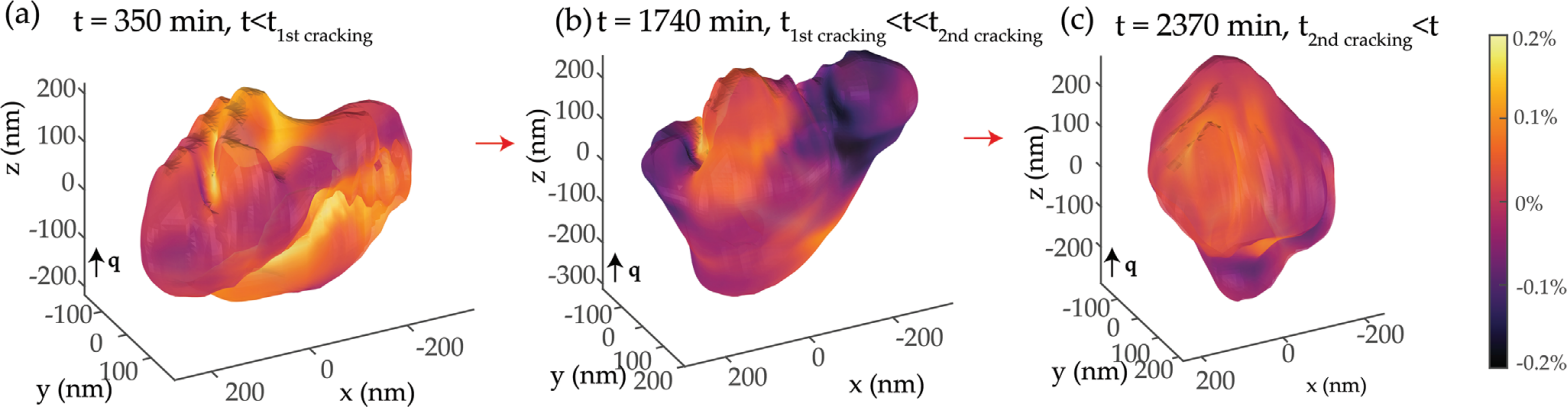
Spatially resolved strain evolution. a) Distribution of strain in the pristine grain (surface at 15% amplitude). b) Distribution of strain in the grain before the main cracking event (surface at 15% amplitude). Note the volumes of different lattice constants visible in the grain. c) Distribution of strain in the grain after cracking (surface at 15% amplitude).

The displacement field provides information about the distribution of the strain in the [110] direction, which is a derivative of the dislocation field along the scattering vector. Analysis of the strain distribution (**Figure** **5**) shows a significant spatial difference in strain accumulation across the grain. In the beginning stages of the process, the strain is distributed homogeneously (Figure 5a), with a variation of ±0.1% of the crystal lattice spacing. However, after the first ≈1500 min, the accumulated strain in the main and detaching volumes of the grain differ by ≈0.4%. More precisely, the average lattice spacing in the volume that detaches after the cracking event seen in Figure 3 is 0.4% lower, signifying either evolving external stress from neighboring grains or a lower penetration by H and O ions. Incorporation of oxygen ion is anticipated to produce a higher impact on the strain.^[^ ^41^ ^]^ Note that the strain difference between the center of the grains and their surface is, in comparison, much smaller (<0.1%), suggesting a more homogeneous ion distribution within the two volumes. Different lattice constants induced in the separating volumes before facet formation, suggesting different H^+^ concentrations, lead us to speculate, therefore, that the non‐equilibrium effects and the interaction with the neighboring grains make {112} termination plane energetically more favorable.

## 3 Conclusions

In summary, we reveal the in situ nanostructure dynamics in a proton‐conducting solid oxide by applying coherent X‐ray diffractive imaging to sintered BZY during hydration at 200 °C. gBCDI fills a method gap for studying the nanoscale dynamics of the crystal lattice in electrolyte candidates for protonic ceramic electrochemical devices in situ. We found unexpectedly active defect nucleation and grain boundary changes at 200 °C. Our results reveal the abundance of newly generated, mobile dislocations that align preferentially along the {110} planes and the cracking of the grains producing uncommon facets. Imaging shows cracking of the grains along the {211} crystallographic planes, generating facets energetically unfavorable in equilibrium conditions. The cracking occurs in the vicinity of the mobile dislocations, suggesting strong interaction between defects. Given that protonic ceramic electrochemical cells commonly operate at even higher temperatures of 300–600 °C, the observed crystal lattice dynamics on the nanoscale and grain instability of BZY at 200 °C in the absence of electric current merit further investigation of effects of nanostructure dynamics on stability, hydration, and proton transport in other PCCs. Future investigations with fuel, in H_2_ atmosphere, will also be necessary. Electrolytes sintered at higher temperatures (1600 °C^[^ ^42^, ^43^ ^]^) also present an interesting avenue of investigation due to a differing density and possibly mechanics. Our results suggest a loss mechanism of active material during temperature and humidity cycling, for example, during the start‐up and shut‐down cycles. The lost material will not be afterward able to participate in the electrochemistry. Operando measurements are required to better quantify the connection between degradation and functional properties. Furthermore, we found the formation of clearly distinct regions in individual grains with different lattice constants, which together with the changes in the interfacial surface to volume ratio means that topological defects affect hydration in electrochemical devices on the timescale of hours, not just as a direct transport channel, but also through the surrounding lattice changes. Thus, submicron structure and mechanical properties in solid oxide proton conductors impact not just the long‐term structural stability and statically proton conduction, but give rise to a dynamic, constantly evolving system, further changing the functionality of the ceramic electrolyte. Mesostructure, external strain, superior mechanical properties, and other methods to control the evolution of non‐equilibrium defects and grain boundaries are, therefore, essential to the advancement of protonic ceramic electrochemical devices.

## 4 Experimental Section

### Synthesis of the BaZr_0.8_Y_0.2_O_3‐d_ Pellets

The BZY pellets were prepared from crystalline BZY powders, which were first formed from nitrate precursors via a sol–gel synthesis followed by calcination at 900 °C for 5 h in the air (3 °C min^−1^ heating rate). Subsequently, the crystalline powders were pressed into ≈50 mm diameter and 50 µm thick pellets (see Figure S1, Supporting Information) and heated them at 1100 °C for 36 h in the air (1 °C min^−1^ heating and cooling rate) to sinter the grains. XRD and surface electron microscopy characterization are presented in Figure S1, Supporting Information. The pellets were later broken into smaller (≈1–10 mm diameter) plates for BCDI measurements due to brittleness.

### Details of the In Situ X‐Ray Coherent Diffraction Measurements

The in situ coherent X‐ray measurements were performed at the beamline 34 ID‐C of the Advanced Photon Source (Argonne National Laboratory, ANL, USA), at a photon energy of 9 keV and sample–detector distance of 1 m. Timepix (34ID) 2D detector with a pixel size of 55 µm × 55 µm was used. Water vapor content inside the in situ chamber was estimated as 30 g m^−3^ assuming water vapor saturation at room temperature from the method used (bubbling). This was supported by condensation appearing in the chamber after cooling. (110) Bragg diffraction peaks were collected (scattering angle 26.6 degrees) from individual grains in the sintered BZY pellet for over 30 h at 200 °C in a humid nitrogen atmosphere (setup scheme in Figure 1a). At every new time point the sample was realigned to ensure that a single Bragg peak is measured, corresponding to a single grain. No multiple intersecting peaks, which would mean multiple grains, were observed. Collecting full 3D reciprocal space maps of the Bragg diffraction peaks required 1–3 min of rocking the sample chamber in the scattering plane (schematically shown in Figure 1b). A full Bragg peak angular spread was below 1 degree, and an angular step below 0.01 degree was required to sufficiently oversample the speckle pattern for phase retrieval.^[^ ^31^ ^]^ Bragg diffraction peaks from individual grains remained stable over hours in a pure nitrogen atmosphere at 200 °C without introducing humidity, therefore excluding significant radiation damage effects on the in situ measurement.

### Phase Retrieval Procedure

Phase retrieval combined the error‐reduction (ER) algorithm alternating with hybrid input–output (HIO) algorithm in 50/10 combination. Retrieval was performed without binning, as sufficient signal and resolution were achieved. Iteration number was settled at 610. All attempts resulted in very similar reconstructions. We used an average of five results in this work, each being an average of 20 best reconstructions retrieved in a guided procedure developed in ref. [6] (8 generations, 40 population). The nanoparticle shape was found by averaging the amplitudes of the reconstructions and applying a threshold of 15% (higher than the threshold during retrieval) to that average amplitude.^[^ ^5^ ^]^ The reconstructions were run using a GPU optimized code on multiple GeForce 1080 and 2080 graphics cards.

### Statistical Analysis

Operando BCDI measurements represented a result of phase retrieval (involving Fourier transform) on a unique, unrepeatable measurement of a crystal grain condition at a certain moment in time. Nevertheless, wherever possible (Figure 2) an error estimate for the values derived directly from intensity measurements was provided. The measure of error in integral peak intensity was estimated by comparison with a measurement performed with small delay (2 min), as a difference. The measure of error in peak width (standard deviation *σ* of the intensity distribution) was estimated according to ref. [33] through the fourth central moment of the intensity distribution as $\sqrt[{4}]{\mu_{4}-\sigma^{4}}$.

## Conflict of Interest

The authors declare no conflict of interest.

## Supporting information

Supporting Information

## Data Availability

The data that support the findings of this study are available from the corresponding author upon reasonable request.
