# Supplementary material for: In Situ Nanoscale Dynamics Imaging in a Proton‐Conducting Solid Oxide for Protonic Ceramic Fuel Cells
Source: Adv Sci (Weinh). 2022 Jun 24;9(25):2202096. doi: 10.1002/advs.202202096 (PMC9443464; doi:10.1002/advs.202202096)
Supplement: Supplementary file 1 — Supporting Information [file ADVS-9-2202096-s001.pdf]

ADVANCED SCIENCE
Open Access

Supporting Information

for *Adv. Sci.*, DOI 10.1002/advs.202202096

In Situ Nanoscale Dynamics Imaging in a Proton-Conducting Solid Oxide for Protonic Ceramic Fuel Cells

Oleg Gorobtsov, Yumeng Song, Kevin Fritz, Daniel Weinstock, Yifei Sun, Dina Sheyfer, Wonsuk Cha, Jin Suntivich and Andrej Singer*

Supporting Information

In-situ nanoscale dynamics imaging in a proton-conducting solid oxide for protonic ceramic fuel cells

Oleg Yu. Gorobtsov†*, Yumeng Song†, Kevin Fritz†, Daniel Weinstock†, Yifei Sun†, Dina Sheyfer‡, Wonsuk Cha‡, Jin Suntivich†, Andrej Singer†

S1. Pre-characterization of the $BaZr_{0.8}Y_{0.2}O_{3-d}$ pellet

Laboratory x-ray diffraction (XRD) patterns for powder and pellet can be indexed to BaZrO3 (matching International Centre for Diffraction Data PDF 01-089-2486) (Figure S1, a) SEM images indicate that the pellet has significant porosity, consistent with the low sintering

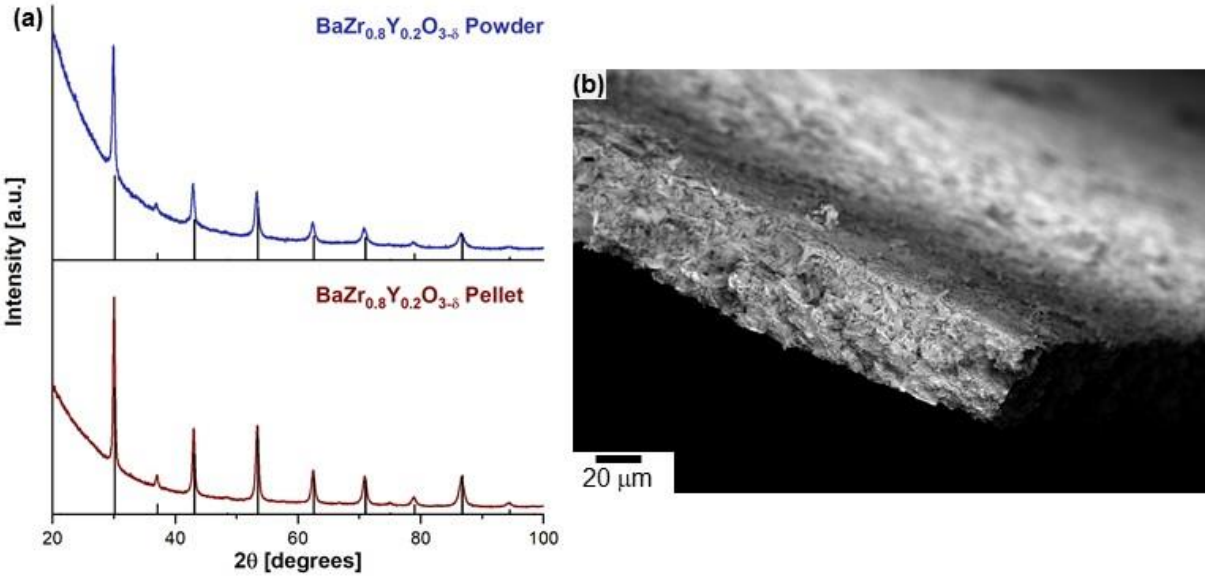

temperature (Figure S1, b).

Figure S1. Pellet characterization. a, Lab-scale XRD pattern for $BaZr_{0.8}Y_{0.2}O_{3-d}$ powder and $BaZr_{0.8}Y_{0.2}O_{3-d}$ pellet matches with PDF 01-089-2486, **b,** Cross-sectional SEM image of $BaZr_{0.8}Y_{0.2}O_{3-d}$ pellet
